# The COMET Initiative database: progress and activities from 2011 to 2013

**DOI:** 10.1186/1745-6215-15-279

**Published:** 2014-07-10

**Authors:** Elizabeth Gargon, Paula R Williamson, Douglas G Altman, Jane M Blazeby, Mike Clarke

**Affiliations:** 1 University of Liverpool, Department of Biostatistics, 1st floor Duncan Building, Daulby Street, Liverpool L69 3GA, UK; 2 Centre for Statistics in Medicine, University of Oxford, Oxford OX3 7LD, UK; 3 School of Social and Community Medicine, University of Bristol, Canynge Hall, 39 Whatley Road, Bristol BS8 2PS, UK; 4 Institute of Clinical Sciences, Block B, Queens University Belfast, Institute of Clinical Sciences, Block B, Royal Victoria Hospital, Grosvenor Road, Belfast BT12 6BA, UK

**Keywords:** Core outcome set, Database, Resources

## Abstract

The Core Outcome Measures in Effectiveness Trials (COMET) Initiative database is an international repository of studies relevant to the development of core outcome sets. By the end of 2013, it included a unique collection of 306 studies. The website is increasingly being used, with more than 12,000 visits in 2013 (a 55% increase over 2012), 8,369 unique visitors (a 53% increase) and 6,844 new visitors (a 48% increase). There has been a rise in visits from outside the United Kingdom, with 2,405 such visits in 2013 (30% of all visits). By December 2013, a total of 4,205 searches had been completed, with 2,139 in 2013 alone.

## Correspondence/findings

### Background

In January 2014, *The Lancet* published a series of papers addressing the important issue of waste in research that may be due to important outcomes not being assessed [1] or to selective reporting of outcomes [2]. Selective reporting or nonreporting of studies and outcomes may mean that decisions made about health care and research design may not be fully informed, which in turn may lead to wasting of valuable health-care resources. The Core Outcome Measures in Effectiveness Trials (COMET) Initiative was set up to try to improve the usefulness of outcomes in research and to tackle these problems [3]. COMET brings together people interested in the development, reporting and application of core outcome sets (COSs). These sets represent the minimum that should be measured and reported in all clinical trials of a specific condition and are also suitable for use in other types of research and clinical audit [4]. The expectation is that the core outcomes will always be collected and reported and that researchers might also include other outcomes of particular relevance to their specific study. The existence or use of a COS does not imply that outcomes in a particular trial should be restricted to those in the relevant set. The use of COSs will make it easier for the results of trials to be compared, contrasted and combined as appropriate, thereby reducing waste in research.

The members of the COMET Initiative aim to collate and stimulate relevant resources (both applied and methodological), to facilitate the exchange of ideas and information and to foster methodological research in the area of COSs by bringing relevant material together, thus making it more accessible. For COSs to be an effective solution, they need to be easily accessible by researchers and other key groups. To date, however, it has been difficult to identify COSs because they are hard to find in the literature. This may mean that they are not used in new studies or that there is unnecessary duplication of effort in developing new COSs. The COMET Initiative seeks to tackle this problem and to reduce the possibility of waste in research by bringing these resources together in one place [5]. The types of studies included in the database are those in which COSs have been developed, as well as studies relevant to COS development, including systematic reviews of outcomes and patients’ views. Individuals and groups who are planning or developing a COS, who have completed one or who have identified one in an *ad hoc* way can submit it for inclusion. The website and database were launched in August 2011, and in this letter we outline activities and progress related to the COMET website and database up to 31 December 2013 (source of data usage: Google Analytics). We provide data on the value and use of the COMET materials, as well as interest in COSs, above and beyond what might be gleaned from information on, for example, the citation of key articles.

### Activity and content

**Figure 1:**
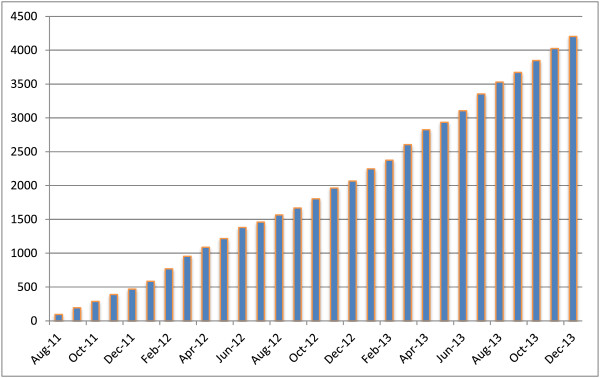
Cumulative number of completed searches in the Core Outcome Measures in Effectiveness Trials (COMET) Initiative database.

On 31 December 2013, a total of 306 studies relevant to the development of COS were included in the COMET database, up from 189 at the end of the previous year. The total at the end of 2013 included 48 planned and ongoing studies, up from 23 at the end of 2012. Since the launch of the database, there has been a rapid increase in the number of users of the website and database. Usage statistics show that the number of visits increased from 7,982 during 2012 to 12,332 in 2013, a 55% increase. The number of unique visitors similarly increased (by 53%), from 5,471 in 2012 to 8,369 in 2013, and the number of new visitors also increased (by 48%), from 4,611 in 2012 to 6,844 in 2013. Full details of the breakdown of these statistics are provided in Table 1. Usage statistics show that, from inception to 31 December 2013, there were almost 100,000 page views, with a 66% increase seen from 2012 to 2013 (from 32,117 to 53,226 page views). By December 2013, a total of 4,205 searches of the database had been run, with 2,139 in 2013 alone—an increase of one-third compared to 1,597 searches in 2012. Cumulative totals are shown in Figure 1. This growth in use suggests that the COMET website and database are gaining interest, acceptance and prominence and that there is a growing awareness of the COMET Initiative.

**Table 1:** Usage statistics, 2011 through 2013

	**Number of visits**			**Number of unique visitors**			**Number of new visitors**		
**Month**	**2011**	**2012**	**2013**	**2011**	**2012**	**2013**	**2011**	**2012**	**2013**
**January**	–	670	1069	–	450	657	–	385	542
**February**	–	762	1017	–	463	648	–	378	525
**March**	–	649	1238	–	429	761	–	358	617
**April**	–	683	1050	–	466	678	–	395	564
**May**	–	659	1088	–	407	721	–	330	504
**June**	–	435	1403	–	305	887	–	260	703
**July**	–	472	945	–	314	650	–	241	526
**August**	804	457	833	503	324	576	494	273	480
**September**	448	483	901	314	347	623	286	288	524
**October**	460	669	984	295	516	802	258	441	689
**November**	686	1117	966	484	854	727	437	757	619
**December**	580	926	838	409	596	639	363	505	551
Totals	**2,978**	**7,982**	**12,332**	**2,005**	**5,471**	**8,369**	**1,838**	**4,611**	**6,844**

The majority of visits to the website were direct or via a search engine. Sixteen percent of all visits were referrals, including Twitter (14%), MRC Network of Hubs for Trials Methodology Research (7%), the Cochrane Collaboration (The Cochrane Library (5%) and the http://www.cochrane.org website (4%)), the EQUATOR Network (3%), the National Institute for Health Research (2%), *BMJ* blogs (2%) and the SPIRIT statement website (2%). Analyses of the website data show that half of the visitors went beyond the page on which they landed and that the most common interaction in both 2012 and 2013 was to complete a search in the COMET database. Other first interactions included moving to the page that provides an overview of the COMET Initiative, accessing the database but without completing a search and visiting the pages containing details of the COMET II or COMET III meeting or collated resources about COMET.

**Figure 2:**
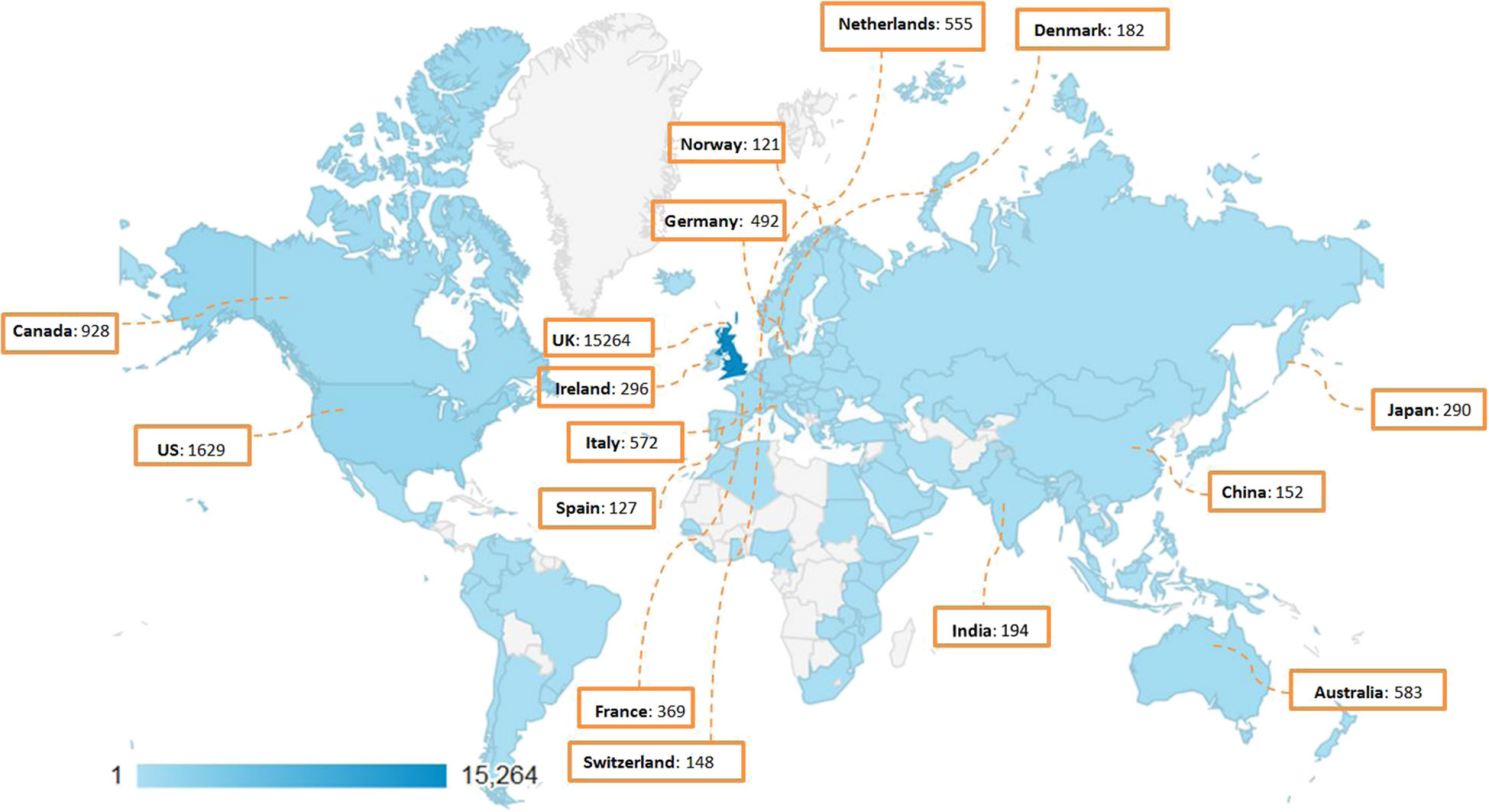
Visits to the Core Outcome Measures in Effectiveness Trials (COMET) Initiative website by country from August 2011 to December 2013, highlighting those with 100+ visits.

The number of countries in which visitors accessed the website increased from a total of 66 in 2011, to 93 in 2012 and to 113 in 2013. This increase in the international usage of the website and database is also reflected in the number of visits. In 2012, 70% of the visits (*n* = 5,577) were in the United Kingdom, 10% were in the United States and Canada (n = 757) and the remaining 20% were in other countries. In 2013, the percentage of visits in the United Kingdom decreased to 59% (7,256 of the 12,332 total visits), increased to 12% in the United States and Canada and increased to an aggregate 29% in all other countries. Figure 2 shows the global distribution of total visits up to 31 December 2013. This increase in the number of visits in countries outside the United Kingdom reinforces COMET as an international initiative and demonstrates increased global awareness of and interest in COSs and the COMET Initiative. Currently, all content and materials available on the website are provided in the English language only.

As noted above, a total of 4,205 searches were completed in the database from its launch in August 2011 to December 2013. The search allows the user to take a structured approach to finding COS. The most frequently applied search criteria were disease category (81%), disease name (69%), status of COS (53%), methods used (29%), stakeholders involved (24%) and type of intervention (20%). The most commonly searched terms were *cancer* (*n* = 256), *consensus* (*n* = 218) and *mental health* (*n* = 204).

### Plans for the future

Several processes are underway to ensure that the COMET database is comprehensive and up-to-date. Individuals and groups who are planning or developing a COS can continue to submit it for inclusion. A systematic review designed to identify studies was recently completed and published [6]. This review involved extensive searches of the health literature to identify studies in which the investigators sought to determine which outcomes or domains to measure in all clinical trials of a specific condition. Studies that were not already included in the COMET database are being added to it (and thus did not contribute to the number of studies reported here), and an annual search of the literature will take place to keep the database current. We are also planning a review of methodological papers to include in the COMET database. The content of the website will continue to be updated regularly, and we plan to extend the patient and public involvement resources on the website beyond the plain language summary that is currently available. We will continue to increase awareness outside the United Kingdom at meetings in Europe and the United States. The next COMET meeting (COMET IV) will be held in Rome later this year. Growing awareness of the need for COSs and increased knowledge of the COMET Initiative should continue to be reflected in the website and database usage figures, which will continue to be monitored and assessed annually.

## Abbreviations

COMET: Core Outcome Measures in Effectiveness Trials; COS: Core outcome set(s).

## Competing interests

DA, JB, MC and PW are members of the COMET Management Group and coapplicants for grants to support COMET and related work. EG is a member of the COMET Management Group and is the COMET Project Coordinator.

## Authors’ contributions

EG and MC conceived the idea for the report. EG performed the analysis. EG, PW, MC, JB and DA interpreted data. EG wrote the manuscript with significant input from PW and MC. All authors read and approved the final manuscript.
